# Triple-Negative Breast Cancer: Adjuvant Therapeutic Options

**DOI:** 10.1155/2011/696208

**Published:** 2011-06-21

**Authors:** Ayca Gucalp, Tiffany A. Traina

**Affiliations:** ^1^Breast Cancer Medicine Service, Memorial Sloan-Kettering Cancer Center (MSKCC), New York, NY 10065, USA; ^2^Weill Medical College of Cornell University, New York, NY 10065, USA

## Abstract

Triple-negative breast cancer (TNBC), a subtype distinguished by negative immunohistochemical assays for expression of the estrogen and progesterone receptors (ER/PR) and human epidermal growth factor receptor-2(HER2) represents 15% of all breast cancers. Patients with TNBC generally experience a more aggressive clinical course with increased risk of disease progression and poorer overall survival. Furthermore, this subtype accounts for a disproportionate number of disease-related mortality in part due to its aggressive natural history and our lack of effective targeted agents beyond conventional cytotoxic chemotherapy. In this paper, we will review the epidemiology, risk factors, prognosis, and the molecular and clinicopathologic features that distinguish TNBC from other subtypes of breast cancer. In addition, we will examine the available data for the use of cytotoxic chemotherapy in the treatment of TNBC in both the neoadjuvant and adjuvant setting and explore the ongoing development of newer targeted agents.

## 1. Triple-Negative Breast Cancer: Adjuvant Therapeutic Options

Each year more than 1.3 million new cases of breast cancer are diagnosed worldwide. In spite of numerous advances in prevention, surgical resection, and adjuvant radiotherapy and chemotherapy, it is estimated that approximately 450,000 women will die of this disease globally each year [1]. Triple-negative breast cancer (TNBC), a subtype distinguished by negative immunohistochemical assays for expression of the estrogen and progesterone receptors (ER/PR) and human epidermal growth factor receptor-2 (HER2), represents approximately 15% of all breast cancers. Patients diagnosed with TNBC generally experience a more aggressive clinical course exacerbated by the lack of effective targeted therapies. Moreover, despite best available therapy, TNBC accounts for a disproportionate number of breast cancer-related deaths, further highlighting the need for novel therapeutic approaches for the management of this high-risk subset of patients [2–4]. In this paper, we will review the epidemiology, risk factors, prognosis, and the molecular and clinicopathologic features that distinguish TNBC from other subtypes of breast cancer. In addition, we will examine the available data for the use of cytotoxic chemotherapy in the treatment of TNBC in both the neoadjuvant and adjuvant setting and explore the ongoing development of newer targeted agents. 

## 2. Clinicopathologic and Molecular Features of TNBC

Human breast cancers represent a heterogenous disease group characterized by varied clinical presentations and responses to therapy. In the past decade, the use of complementary DNA (cDNA) microarrays has furthered our understanding of the underlying biologic diversity of these tumors well beyond the identification of hormone receptor and HER2 status, to include distinct gene expression profiles which correlate with disease progression and clinical outcomes. 

Perou, Sørlie, and colleagues have identified 5 molecularly distinct gene expression profiles that may one day allow for clinically relevant classification of breast cancer [5–7]. This diversity is apparent within the triple-negative subgroup as well, evidenced by the identification of multiple molecular profiles which demonstrate low expression of ER, PR, and HER2 including the basal-like, claudin-low, and molecular apocrine/ER(−) class A subtypes [8–10]. One such group, the basal-like breast cancers (BLBC), expresses minimal levels of ER/PR/HER2 and high levels of CK 5/6, CK 14, CK 17, p-cadherin, caveolin-1, carbonic anhydrase IX gene (CA IX), p63 (a member of the p53 family of transcription factors and a myoepithelial stem cell regulator), and epidermal growth factor receptor (EGFR or HER1) similar to their cell of origin in normal breast tissue [11]. Although not completely identical, basal-like and triple-negative breast cancers share numerous molecular features with up to 70% concordance between the two subgroups [12–15]. Interestingly, tumors associated with germline mutations in BRCA-1 demonstrate a significant overlap in their clinical and molecular presentation with basal-like tumors [16, 17]. BRCA-1-associated tumors are generally triple-negative [18, 19] and cluster alongside the basal-like tumors on microarray [7] with a significant proportion expressing CK 5/6, 14, 17, p-cadherin, and EGFR [17, 19–22]. Studies that have reviewed the histological presentation of TNBC and BLBC demonstrate that >90% of these tumors arise from the breast ducts and are often associated with higher nuclear and histologic grade, high mitotic index, and more aggressive phenotypic features [2, 13, 19, 23–25]. 

## 3. Epidemiology

Epidemiologic studies demonstrate that women diagnosed with TNBC manifest a significantly different set of clinicopathologic features and risk factors when compared to women with other subtypes of breast cancer. TNBC comprises approximately 15% of all breast cancers diagnosed; however, in certain select populations, the prevalence may be higher, for example, among premenopausal African American and Hispanic patients [4, 23, 25–29]. Based on multiple population-based studies, women with TNBC on average are younger at diagnosis and have disease associated with both modifiable and nonmodifiable risk factors including earlier age at menarche and at first pregnancy, increased parity, decreased breastfeeding, higher BMI, and lower socioeconomic status [2, 25, 26, 28, 30–36].

## 4. Patterns of Recurrence and Prognosis

Population-based studies have confirmed the increased rate of breast cancer-related deaths among patients with TNBC and have identified distinct patterns of recurrence for this subgroup [2, 23]. Patients diagnosed with TNBC have a higher likelihood of recurrence within the first three years of diagnosis and death from disease within the first five years [2, 37]. Additionally, once metastatic disease has been identified, patients with TNBC and BLBC experience shorter survival times in comparison to patients with other tumor subtypes [2, 24, 27]. Among TNBC patients, recurrences beyond 5 years are less common and at 10 years, overall survival rates among the varying subgroups are roughly equivalent [38]. 

Women with TNBC more often develop visceral versus osseous metastases when compared to their hormone receptor-positive counterparts [4, 39]. In a large multicenter study which included >2000 patients with TNBC, Lin and colleagues demonstrated that women with TNBC were more likely to develop lung (Odds Ratio (OR) 2.27, 95% confidence interval (CI) 1.50, 3.43; *P* = .0001) or brain metastases (OR 5.32, 95% CI 2.85, 9.91; *P* < .0001) as their first site of recurrence. In comparison, these women demonstrated a much lower risk of bone recurrence (OR 0.23, 95% CI 0.16, 0.33; *P* < .0001) [25]. Numerous studies have demonstrated an increased rate of CNS metastases in women with TNBC [40–42]. In a large single-institution retrospective analysis, 1,138 women with stage I-III TNBC were identified, of which 29% had developed recurrence at median five-year followup. Of those with documented recurrence, 21% had developed brain metastases. Median survival for those with brain metastases was 25 weeks with survival rates at 6 months and 12 months of 48% and 25%, respectively [43]. Similar results were seen in other studies and when compared to patients with phenotypically different breast cancers, women with TNBC experienced shorter median survival after diagnosis of CNS involvement [41, 44]. 

## 5. Therapeutic Options

### 5.1. Chemotherapy

To date, many studies have examined the utility of traditional chemotherapy for the treatment of patients with TNBC and have confirmed the benefits of these agents in both the adjuvant and neoadjuvant settings. A meta-analysis from the Early Breast Cancer Trialists' Collaborative Group (EBCTCG) was one of the first reviews to determine the efficacy of polychemotherapy in the treatment of ER-poor individuals. Over 6000 women with ER-poor breast cancer, treated in 46 separate randomized trials of adjuvant polychemotherapy (CMF X6 45%; FAC or FEC X6 31%, other 24%) in the prepaclitaxel era were examined. At ten-years followup, the women treated with polychemotherapy demonstrated a significantly reduced risk of recurrence (age <50 hazard ratio (HR) 0.73, age 50–69 HR 0.82) as well as both breast cancer-related (age < 50 HR 0.73, age 50–69 HR 0.86) and all-cause mortality (age < 50 HR 0.75, age 50–69 HR 0.87) [45]. As many of these trials were initiated prior to uniform HER2 testing, information regarding the true proportion of TNBC in the trial population remains unknown. Nevertheless, the results of this large meta-analysis strengthened the hypothesis that improved outcomes could be achieved in this high-risk population with the use of multiple chemotherapeutic agents. 

Similarly, Berry and colleagues completed a retrospective analysis regarding the efficacy of adjuvant chemotherapy in relation to ER status among women enrolled in three adjuvant chemotherapy trials coordinated by the Cancer And Leukemia Group B (CALGB) and the US Breast Intergroup (CALGB 8541, 9344/INT 1048, 9741/INT C9741). In comparison to the women with ER-positive disease, the women with ER-negative tumors treated with regimens which included higher doses, taxanes, and dose-dense (dd) scheduling fared better in terms of risk of recurrence and overall survival. When examined in total, ER-negative women who received dd doxorubicin, cyclophosphamide followed by paclitaxel (AC→T) compared to low-dose cyclophosphamide, doxorubicin, and 5-fluorouracil (CAF) experienced a 55% (Confidence Interval (CI) 37–68%) relative risk reduction in recurrence. In comparison, women with ER-positive disease experienced a 26% risk reduction, (CI −4–48%). Furthermore, the absolute improvement in disease-free survival (DFS) (22.8% versus 7%  *P* < .001) and overall survival (16.7% versus 4.0%  *P* < .001) incurred by the ER-negative subgroup further underscored the benefits of multidrug chemotherapy regimens in this subgroup [46]. 

When analyzed individually, CALGB 9344 and 9741 not only highlighted the therapeutic benefit of taxanes in the adjuvant setting but also contributed to the observation that ER-negative individuals specifically may experience preferentially improved outcomes from use of taxane-inclusive regimens. Unplanned subset analyses in both of the aforementioned studies demonstrated a trend towards improved risk reduction in terms of recurrence for women with ER-negative disease (9344: 28% versus 9%; 9741: 32% versus 19%) [47, 48]. Further examination of the HER2(−) subgroup, demonstrated that women who were both ER and HER2 negative realized a statistically significant improvement in DFS with the addition of paclitaxel therapy (*P* = .002) whereas ER+ HER2(−) individuals did not experience a similar benefit (*P* = .71), thereby supporting the inclusion of taxanes in adjuvant therapy for the treatment of patients with TNBC [48]. Interestingly, women with HER2+ breast cancer, regardless of hormone receptor status, experienced a statistically significant improvement in terms of DFS with the addition of paclitaxel chemotherapy. However, comparisons between women with ER/PR(−) HER2(−) disease and those with ER/PR(−) HER2(+) disease remain complicated, because of the retrospective nature of the analyses and the increasing use of anti-HER2 therapy. 

Several studies have substantiated the positive impact of chemotherapy in the treatment of patients with TNBC in the neoadjuvant setting as well. Among 1,118 patients treated with neoadjuvant chemotherapy, (>80% treated with anthracycline-based regimen; 53% treated with an additional taxane), patients with TNBC had a significantly higher rate of pathologic complete response (pCR) in comparison to patients with non-TNBC (22% versus 11%; *P* = .034). And despite an overall worse progression-free survival (PFS) and overall survival (OS) among patients with TNBC, those individuals who achieved a pCR had similar overall survival rates as non-TNBC patients with pCR [4]. A retrospective analysis of patients treated predominantly with anthracycline and anthracycline/taxane containing preoperative regimens (91% and 58%, resp.), which included 317 patients with TNBC, demonstrated a similar rate of pCR among this subgroup, 22.4%. Comparatively patients with hormone receptor-negative disease attained significantly higher rates of pCR (24% versus 8%  *P* > .001) than the hormone receptor-positive group. Similar to that shown in the Liedtke trial [4], patients who achieved a pCR also experienced improved PFS and OS [49]. When objective response to neoadjuvant chemotherapy (weekly T × 12 followed by fluorouracil, doxorubicin, cyclophosphamide (FAC) × 4) was examined in relation to the established molecular subtypes of breast cancer, Rouzier et al. identified the highest rates of pCR among the BLBC (45%; CI 24–68%) and erbB2+ (45% CI 23–68%) subgroups. In comparison, of the 30 luminal breast cancers only 2 achieved a pCR (7% CI 1–22%) [50]. Carey et al. demonstrated similar results when patients were treated with 4 cycles of neoadjuvant AC. Furthermore, patients who achieved a complete pCR, regardless of molecular subtype, experienced better outcomes in terms of distant disease-free survival [51]. Despite the varied neoadjuvant regimens studied in these trials and many others, the consistently higher rates of pCR among the TNBC/BLBC subgroup in response to chemotherapy reaffirms the utility of this therapeutic strategy in the treatment of this subgroup.

Many trials support the use of cytotoxic agents for the treatment of patients with TNBC; however, the superiority of one regimen over another has not been clearly established. For example, a retrospective review of the MA5 trial, (adjuvant cyclophosphamide/epirubicin/ fluorouracil (CEF) versus CMF), delineated overall survival in relation to molecular phenotype. Patients with BLBC who received CMF were shown to have a superior 5-year overall survival rate in comparison to those who received the anthracycline-based regimen (71% versus 51%) [52]. In another retrospective review, Colleoni et al. demonstrated that patients with TNBC treated with CMF (either 3 or 6 cycles) experienced the greatest benefit from chemotherapy in terms of relative risk reduction (HR 0.46, CI 0.29–0.73, *P* = .009) when compared to individuals with hormone receptor- and/or HER2-positive disease [53]. Furthermore, review of the literature fails to explain whether response rates to chemotherapy among this subgroup are a result of the efficacy of specific regimens or the increased chemosensitivity of individuals with TNBC. 

More recently, a number of preclinical studies examining the activity of platinum agents in the treatment of TN and BRCA1-associated breast cancers have demonstrated increased sensitivity to these agents. BRCA1-associated tumors are deficient in the genes that encode for proteins critical in DNA integrity, genomic stability, and DNA repair. In preclinical models of BRCA1-deficient breast cancers, there is an increased susceptibility to DNA-damaging agents, particularly those able to induce double-strand breaks such as cisplatin or carboplatin [54–57]. Byrski et al. treated 10 women with BRCA1-associated breast cancer, (9 with known TNBC) with preoperative single-agent cisplatin (75 mg/m^2^ every 3 weeks × 4). All but one patient on the trial achieved a pCR, and she was noted not to have completed all 4 cycles of chemotherapy [58]. Given the small numbers of patient in this trial and the limited followup, it is difficult to draw conclusions regarding reduction in risk of recurrence and survival. However, these data do suggest the activity of platinum agents in this subgroup and warrant further study in prospective trials as detailed below. 

TNBC share numerous clinical, molecular, and pathologic features with BRCA mutation-related breast cancers including altered BRCA function and a high degree of genomic instability as well as impaired DNA damage repair. Consequently, many studies have been initiated to study the efficacy of platinum salts in this subgroup. Silver and colleagues tested the efficacy of neoadjuvant cisplatin in a TNBC population not enriched for BRCA-mutation carriers. Eighteen of the 28 patients experienced a clinical response to therapy demonstrating either a partial or complete response with 6 achieving complete pathologic remission. Two of the 6 patients who attained pCR were germline BRCA1-mutation carriers. As a correlate, levels of BRCA1 mRNA expression and BRCA1 promoter methylation were measured in relation to response to therapy. Both lower levels of BRCA expression as well as BRCA promoter methylation, which is inversely proportional to BRCA expression, were correlated with response to cisplatin therapy suggesting that a subgroup of TNBC patients may demonstrate a “BRCA-like” phenotype which predisposes them to cisplatin-sensitivity [59]. 

Most recently, the BALI-1 trial randomized 173 patients with metastatic TNBC to receive either cisplatin alone versus cisplatin in combination with cetuximab. Final analysis of the trial demonstrated a modest yet statistically significant improvement in PFS among patients who received combination therapy, 1.5 versus 3.7 months (HR 0.675 CI 0.470–0.969, *P* = .032). Notwithstanding the doubling of the overall response rate in the combination arm (10.3% versus 20%), the study failed to meet its primary endpoint of greater than a 20% response among patients who received both cisplatin and cetuximab [60]. This highlights the need for further studies to examine the efficacy of single-agent platinum therapy to treat TNBC as well as the use of targeted therapies, like cetuximab, in an unselected population. 

Numerous trials are currently underway in the adjuvant and neoadjuvant setting to prospectively study the efficacy of polychemotherapy, including combinations with newer chemotherapeutic agents and novel targeted therapies.

- CALGB 40603 is a randomized Phase II trial where patients are enrolled in 1 of 4 arms which include: Arm 1: weekly paclitaxel x12 followed by dd AC x4, Arm 2: Arm 1 + bevacizumab every 2 weeks, Arm 3: Arm 1 + carboplatin, and Arm 4: Arm I + bevacizumab as in arm II + carboplatin as in arm III (NCT00861705).
- A Phase III trial enrolling patients into either docetaxel/anthracycline (epirubicin versus doxorubicin)/cyclophosphamide versus docetaxel and cyclophosphamide is set to assess the added benefit of anthracycline-containing preoperative regimens in TNBC (NCT00912444).
- A randomized Phase III study of standard adjuvant chemotherapy alone or followed by 1 year of metronomic capecitabine (650 mg/m^2^ BID) is underway with the primary endpoint of DFS. (NCT01112826) Thus far, capecitabine has not been studied specifically in the triple-negative population. Additionally, the data which currently exist are based on retrospective subgroup analyses which demonstrated that treatment with capecitabine resulted in limited activity in comparison to standard chemotherapy in the adjuvant setting as well as poorer survival outcomes in comparison to non-TNBC patients in the metastatic setting [61–63].
- A Phase II study of ixabepilone in the neoadjuvant setting demonstrated promising results; in subgroup analysis, patients with TNBC demonstrated a pCR rate of 19% (CI 9–34%) [64]. However, a more recent neoadjuvant Phase II trial randomizing patients to AC followed by ixabepilone versus AC followed by paclitaxel did not demonstrate a significant difference in pCR rates between the two regimens, 34% versus 41% [65]. In light of this, the two adjuvant Phase III trials (PACS08 (NCT00630032) and TITAN (NCT00789581)) initiated to compare ixabepilone directly with more commonly used taxanes have been terminated by Bristol-Myers Squibb.

## 6. Targeted Therapies

### 6.1. Antiangiogenic Agents

Agents that target angiogenesis are appealing for the treatment of TNBC because higher levels of vascular endothelial growth factor (VEGF) and VEGF-2 have been shown in women with TNBC suggesting its potential as a prognostic tool as well as a putative target for therapeutic intervention [66, 67]. Bevacizumab, a humanized monoclonal antibody to VEGF, is approved by the FDA for the treatment of several solid tumors and was granted accelerated approval for the treatment of first-line MBC in combination with paclitaxel [68]. At this time, the approval of bevacizumab and paclitaxel for this first-line indication is under review. 

Bevacizumab has been studied in three randomized Phase III trials in combination with chemotherapy for the first-line treatment of metastatic breast cancer. E2100 randomized >700 women to receive weekly paclitaxel with or without bevacizumab. Women who received bevacizumab experienced a significantly higher objective response rate (36.9% versus 21.2%, *P* ≤ .001) and improvement in PFS (11.8 versus 5.9 months, *P* ≤ .001). Subset analysis of the women with ER/PR(−) disease, the majority of whom were negative for HER2 (>90%), demonstrated a robust prolongation of PFS in comparison to the hormone receptor-positive patients [69]. 

The benefit of bevacizumab for patients with triple-negative MBC was replicated in AVADO, a placebo-controlled study evaluating the addition of bevacizumab (at 2 doses: 7.5 mg/kg or 15 mg/kg) to docetaxel. PFS was significantly improved for those patients who received docetaxel in combination with bevacizumab when compared to women who received docetaxel monotherapy. Median PFS for docetaxel monotherapy in comparison to the bevacizumab_7.5_ and bevacizumab_15_ groups was 8.0 versus 8.7 (HR 0.79 *P* = .03) and 8.8 (HR 0.72 *P* = .001) months, respectively. Unplanned subgroup analysis of the ER/PR/HER2(−) subset revealed PFS values consistent with the study population as a whole (bevacizumab_7.5_: HR 0.83; bevacizumab_15_: HR 0.68) [70]. 

The third Phase III trial of bevacizumab in the first-line setting randomized patients to receive bevacizumab or placebo in combination with several different chemotherapy options (anthracyclines, taxanes, and capecitabine). Based on investigator assessment, the addition of bevacizumab to capecitabine or an anthracycline/taxane resulted in statistically significant prolongation in PFS as compared to placebo (8.6 versus 5.7 months HR 0.69 *P* = .0002 and 9.2 versus 8.0 months HR 0.65 *P* = .0001, resp.) [71]. Further analysis of the ER/PR/HER2-negative subgroup demonstrated a nonsignificant improvement in the median PFS in both the capecitabine (4.2 versus 6.1 months, HR 0.72, CI 0.49–1.06) and anthracycline/taxane cohorts (8.2 versus 14.5 months, HR 0.78, CI 0.53–1.15) [72]. 

Although all three trials failed to demonstrate an OS benefit with the addition of bevacizumab in the metastatic setting, improvements in response rate and PFS were achieved across all subtypes suggesting activity in breast cancer. Furthermore, despite the inherent limitations associated with unplanned retrospective subgroup analyses all three trials demonstrated at least a trend towards improved RR and PFS with the addition of bevacizumab in patients with TNBC. 

Currently, there are multiple Phase II/III trials designed to test the efficacy of bevacizumab in the neoadjuvant/adjuvant setting. Three Phase II studies are currently accruing patients to assess the benefits of including bevacizumab in conjunction with platinum agents in the neoadjuvant setting for patients with TNBC. As previously discussed, CALGB 40603 is a multiarm trial comparing weekly T followed by dd AC with the addition of either bevacizumab or carboplatin alone or in combination. (NCT00861705) The NEAT trial is a single-arm, open-label study of docetaxel/carboplatin in combination with bevacizumab given every 3 weeks for 6 cycles preoperatively. (NCT01208480) In a similar study based at the University of Tennessee Cancer Institute, patients will receive neoadjuvant nanoparticle albumin bound (nab-) paclitaxel (day 1, 8, 15), carboplatin (day 1), and bevacizumab (day 1, 15) over the course of a 28-day cycle × 4 cycles followed by ddAC × 4 in addition to bevacizumab for the first two cycles. In this trial to assess the utility of maintenance bevacizumab, postoperatively patients will receive 8 cycles of bevacizumab given every 2 weeks for a total of 16 doses (NCT00777673). 

In the adjuvant setting, the BEATRICE study randomizes patients with TNBC to either standard adjuvant chemotherapy (anthracycline ± taxane or taxane only) or adjuvant chemotherapy in combination with bevacizumab x1 year to assess the primary endpoint of disease-free survival (NCT00528567). 

Additionally multitargeted small molecule tyrosine kinase inhibitors (TKIs), such as sunitinib and sorafenib, which inhibit numerous targets in the antiangiogenesis pathway, have been evaluated for the treatment of MBC. Unfortunately, these agents have thus far demonstrated modest single-agent activity [73–75] and have failed to improve PFS in two large Phase III trials in combination with chemotherapy, (SUN1064: sunitinib plus docetaxel versus docetaxel; SUN1099: sunitinib plus capecitabine versus capecitabine) [76, 77]. There are currently two Phase I/II studies underway in the neoadjuvant setting which were developed to assess the benefit of platinum/taxane-based chemotherapy in combination with these antiangiogenic TKIs (NCT00887575; NCT01194869).

### 6.2. Poly(ADP-ribose) Polymerase (PARP) Inhibitors

PARP is an essential nuclear enzyme that is involved in the recognition of DNA damage and facilitation of single-strand DNA repair through the base excision repair (BER) pathway. Following detection of a DNA strand break, PARP1, the predominant cellular PARP catalyzes the synthesis and transfer of ADP-ribose polymers to target proteins using NAD+ as substrate. As a result, PARP recruits other repair enzymes and facilitates DNA repair and cell survival. BRCA1 and BRCA2 genes encode for proteins critical for DNA integrity and genomic stability. BRCA1 and BRCA2 proteins are essential for cell division, DNA error control, DNA repair, and apoptosis. In patients with BRCA loss (hereditary mutation), inhibition of PARP induces synthetic lethality which means that DNA damage is irreparable and leads to cell death in homozygote tumor cells, but not in normal tissue heterozygote cells which have one functional BRCA allele [78–84].

 In 2005, Farmer and colleagues showed that BRCAdeficient breast cell lines were extremely sensitive to PARP inhibition [81]. Single-agent PARP inhibitors led to impaired single-strand break (SSB) repair causing double-strand breaks (DSBs) to occur in replicating cells. In BRCA wild-type cells, DSBs are repaired via homologous recombination, but in BRCA mutant cells, this compensatory repair pathway is impaired leading to complex rearrangements, loss of repair mechanisms, and cell death. 

As discussed previously, preclinical tumor models of BRCA-associated breast cancers have demonstrated increased sensitivity to therapies which induce DNA damage such as alkylators and radiation (Table 1) [3, 54–57, 85]. Thus, numerous studies in the metastatic setting have paired PARP inhibitors with agents such as platinums and temozolomide. These clinical trials have shown encouraging activity in several solid tumors with acceptable safety profiles (Table 2). Tutt et al. conducted a Phase II study of single agent olaparib in women with BRCA-associated breast cancer. Patients received olaparib at one of two doses. After an interim analysis, patients in the low-dose cohort who had not progressed were offered the option of dose escalation on study. The results of the trial indicated significant objective response rates of 41% (CI 25–59%) among the cohort receiving 400 mg BID and 22% (CI 11–41%) among the cohort receiving 100 mg BID with limited toxicity. Significant prolongation of median PFS was also demonstrated in both cohorts, (maximal-dose cohort 5.7 months (CI 4.6–7.4), low-dose cohort 3.8 months (CI 4.6–7.4)) [86]. These findings suggest that this approach can induce synthetic lethality in homologous recombination repair-deficient cells in general and BRCA deficient cells in particular.

Given the clinical, histologic, and molecular overlap between BRCA-1-associated tumors and TNBC, multiple investigators have theorized that PARP inhibitors may prove efficacious in this subgroup as well. In a Phase II study O'Shaughnessy and colleagues randomly assigned patients to receive carboplatin and gemcitabine alone or in combination with iniparib, an intravenous PARP inhibitor. The data from this trial showed significant improvement in clinical benefit rate (CBR = CR + partial response + stable disease (SD) ≥6 months; 56 versus 34%, *P* = .01), median PFS (5.9 versus 3.6 months HR 0.59, *P* = .01), and median OS (12.3 versus 7.7 months HR = 0.57, *P* = .01) among those individuals who were treated with iniparib and chemotherapy when compared to chemotherapy alone [87]. This work launched a randomized phase III trial evaluating iniparib in combination with carboplatin and gemcitabine versus chemotherapy alone. A recent press release indicates that the study did not reach significance for its coprimary endpoints of OS and PFS. Similar to results seen in the Phase II trial, the combination of iniparib and chemotherapy in the 2nd and 3rd line settings were reported to demonstrate an increase in OS and PFS. The data from this trial have yet to be fully presented, and, thus, the role for this agent and other PARP inhibitors for the treatment of metastatic triple-negative breast cancer remains unclear [88].

Trials incorporating PARP inhibitors, alone or with concomitant cytotoxic agents, are currently being developed in the neoadjuvant/adjuvant BRCA-associated and TNBC populations. Two Phase II trials are currently accruing patients in the neoadjuvant setting either in combination with platinum agents or taxanes. (NCT00813956; NCT01204125) An adjuvant Phase II trial is underway which randomizes patients with residual disease after nonplatinum-based neoadjuvant chemotherapy and definitive surgery to receive either cisplatin alone or in combination with PF-01367338. (NCT01074970).

### 6.3. EGFR Inhibitors

EGFR is expressed in approximately 60% of TNBC [89]. Preclinical work in known TNBC cell lines demonstrated a synergistic decline in proliferation when EGFR TKIs were combined in vitro with either docetaxel or carboplatin. In contrast, as single agents, both erlotinib, a TKI targeting EGFR, and cetuximab, a monoclonal antibody to EGFR, demonstrated minimal single-agent activity [90]. Clinically, EGFR inhibitors have thus far been studied in the metastatic setting. TBCRC 001, a randomized Phase II multicenter trial, examined sequential cetuximab followed by carboplatin at the time of progression versus concurrent cetuximab/carboplatin in patients with pretreated TNBC. Given the poor single agent response rate to cetuximab in the sequential arm, this arm of the trial was closed to accrual early. Those patients receiving cetuximab in conjunction with carboplatin demonstrated a response rate of 18% and a clinical benefit (partial response or SD ≥ 6 months) of 27%. Nevertheless, a majority of patients progressed rapidly on both arms with a reported mean PFS of 2.0 months in the study [91]. A second randomized Phase II study enrolled patients in either a chemotherapy only arm where they received irinotecan/carboplatin (Day 1, 8) versus a combination arm of cetuximab and chemotherapy. Among patients with TNBC, preliminary data from this trial suggests improved response rates in the combination cetuximab and chemotherapy arm (39% versus 19%). However no significant improvement in PFS or OS was reported in any of the subgroups and increased toxicity resulted in dose reductions for both study arms [92]. The BALI-1 trial, as reviewed earlier, failed to meet its prespecified endpoint but did suggest the activity of cetuximab in combination with cisplatin in patients with TNBC. 

Two Phase II studies are currently open to test the efficacy of cetuximab in combination with preoperative chemotherapy, ixabepilone (NCT01097642) and docetaxel (NCT00600249). A neoadjuvant study is accruing patients to assess the pCR rate of erlotinib in combination with chemotherapy. A second component of this trial involves the addition of maintenance erlotinib X1 year after the completion of the patient's adjuvant regimen. (NCT00491816). 

## 7. Conclusion

Currently, standard chemotherapy remains the cornerstone of treatment for patients with TNBC in the preoperative and adjuvant settings. The development of newer biologic and targeted therapies, such as antiangiogenic agents, EGFR inhibitors, and PARP inhibitors, continues to be a promising area of research. Trials are ongoing to assess the efficacy of specific chemotherapeutic regimens alone or in combination with newer targeted agents in both the neoadjuvant and adjuvant setting and will potentially provide the basis for practice-altering changes in our management of this high-risk population. Ideally, clinically appropriate patients with TNBC should be counseled about the availability of ongoing clinical trials and whenever possible be treated within the context of a research study.

## Figures and Tables

**Table 1:** Clinicopathological Features of TNBC versus BLBC versus BRCA-associated Breast Cancer.

Characteristics	Triple-negative breast cancer	Basal-like breast cancer	BRCA-associated breast cancer
Receptor status	Negative	Negative	Often negative
HER2 Status	Negative	Negative	Negative
Cytokeratin 5/6, 17	Often positive	Positive	Often positive
EGFR expression	Often positive	Often positive	Often positive
p53	Often mutated	Often mutated	Often mutated
BRCA status	May be dysfunctional secondary to diminished expression or increased expression of negative regulators of the BRCA pathway	May be dysfunctional secondary to diminished expression or increased expression of negative regulators of the BRCA pathway	Inactivated secondary to a hereditary mutation
Molecular gene expression profile	Often basal-like	Basal-like	Often basal-like
Histology	Ductal/poorly differentiated/high grade	Ductal/poorly differentiated/high grade	Ductal/poorly differentiated/high grade

**Table 2:** Neoadjuvant/Adjuvant clinical trials for patients with triple-negative breast cancer*.

NCI ID	Status	Primary location	Study type	Setting adjuvant	Stage	Regimen
*Chemotherapy*						
NCT00861705	Recruiting	Miriam Hospital (Providence, RI)	Phase II	Neoadjuvant	II-III	Arm A: Paclitaxel D1 weekly × 12 weekly → ddAC D1 × 4 cycles
						Arm B: Arm A + Bevacizumab q2wks (weeks 1, 3, 5, 7, 9, 10, 11, 13, 15, 17)
						Arm C: Arm A + Carboplatin q3wks (wks 1, 4, 7, 10
						Arm D: Arm A + Bevacizumab as in Arm B + Carboplatin as in Arm C.
NCT00912444	Recruiting	Shanghai Jiao Tong University School of Medicine (Shanghai, China)	Phase III	Neoadjuvant	T2N1 OR T3-4/N0-3 OR T0-4/N2-3	Arm A: Docetaxel 75 mg/m^2^ & Doxorubicin 50 mg/m^2^ OR Epirubicin 60 mg/m^2^ & Cyclophosphamide 500 mg/m^2^ D1 × 6 cycles (cycle = 21 days)
						Arm B: Docetaxel 75 mg/m^2^ & Cyclophosphamide 500 mg/m^2^ D1 × 6 cycles (cycle = 21 days)
NCT01112826	Recruiting	Sun Yat-sen University Cancer Center (Guangzhou, China)	Phase III	Adjuvant	T1c-T3, pN0-2	Arm A: Standard adjuvant chemotherapy followed by capecitabine 650 mg/m^2^ BID × 1 yr
						Arm B: standard adjuvant chemotherapy
NCT00789581	Active/Not recruiting	Sarah Cannon Research Institute (Nashville, TN)	Phase III	Adjuvant	Node negative T1c-T3 OR Node positive pN1mi -N2b)	Arm A: Doxorubicin 60 mg/m^2^ & Cyclophosphamide 600 mg/m^2^ D1 × 4 cycles (cycle = 21 days) → Ixabepilone at 40 mg/m^2^ D1 × 4 cycles (cycle = 21 days)
						Arm B: Doxorubicin 60 mg/m^2^ & Cyclophosphamide 600 mg/m^2^ D1 × 4 cycles (cycle = 21 days) → Paclitaxel at 80 mg/m^2^ D1 weekly × 12 weeks
NCT00630032	Active/Not recruiting	Centre Regional Rene Gauducheau (Nantes-Saint Herblain, France)	Phase III	Adjuvant	Node-positive disease OR node-negative disease: II-III OR pT1-4	Arm A: Epirubicin & 5-Fluorouracil & Cyclophosphamide D1 × 3 cycles (cycle = 21 days) → Docetaxel D1 × 3 cycles (cycles = 21 days)
						Arm B: Epirubicin & 5-Fluorouracil & Cyclophosphamide D1 × 3 cycles (cycle = 21 days) → Ixabepilone D1 × 3 cycles (cycles = 21 days)
NCT01216111	Available	Fudan University (Shanghai, China)	Expanded Access	Adjuvant	I-IIIA	Paclitaxel 100 mg/m^2^ & Cisplatin AUC = 2 D1, 8, 15 × 6 cycles (cycle = 28 days)
NCT01276769	Recruiting	Cancer Institute Hospital/Chinese Academy of Medical Sciences (Beijing, China)	Phase II	Neoadjuvant	IIa-IIIc (no T4 disease)	Arm A: Paclitaxel 175 mg/m^2^ D3 & Epirubicin 75 mg/m^2^ D 1, 2 × 2–6 cycles (cycle = 21 days)
						Arm B: Paclitaxel 175 mg/m^2^ D1 & Carboplatin AUC = 5 D2 × 2–6 cycles (cycle = 21 days)
NCT01216124	Available	Fudan University (Shanghai, China)	Expanded Access	Neoadjuvant	I-IIIA	Docetaxel 75 mg/m^2^ D1 & Oxaliplatin 130 mg/m^2^ D2 × 6 cycles (cycle = 21 days)
NCT01238133	Recruiting	Arthur G. James Cancer Hospital and Richard J. Solove Research Institute at Ohio State University Comprehensive Cancer Center (Columbus, OH)	Phase I	Neoadjuvant	II-III	RO4929097 D1-3, 8-10, 15-17 & Paclitaxel D1, 8, 15 & Carboplatin D1 × 6 cycles (cycle = 21 days)
NCT01167192	Recruiting	Washington University School of Medicine (St. Louis, MO)	Phase II	Neoadjuvant	T2-T4, any N	Cisplatin 75 mg/m^2^ IV or Carboplatin AUC = 6 IV, at physician discretion) + XRT × 6 weeks (50–60 Gy to breast/CW; 45–50 Gy to internal mammary nodes, supraclavicular fossa nodes and axillary nodal basins)

*Antiangiogenic agents*						
NCT00861705	Recruiting	Miriam Hospital (Providence, RI)	Phase II	Neoadjuvant	II-III	Arm A: Paclitaxel D1 weekly × 12 weekly → dd AC D1 × 4 cycles
						Arm B: Arm A + Bevacizumab q2wks (weeks 1, 3, 5, 7, 9, 10, 11, 13, 15, 17)
						Arm C: Arm A + Carboplatin q3wks (wks 1, 4, 7, 10
						Arm D: Arm A + Bevacizumab as in Arm B + Carboplatin as in Arm C.
NCT01208480	Recruiting	Severance Hospital (Seoul, Korea)	Phase II	Neoadjuvant	II-III	Bevacizumab & Docetaxel & Carboplatin D1 × 5 cycles (cycle = 21 days → Docetaxel & Carboplatin C6D1
NCT00777673	Recruiting	University of Tennessee Cancer Institute (Memphis, TN)	Phase II	Neoadjuvant	T2-T3, cN1-cN2a	Nab-paclitaxel D1, 8, 15 & Carboplatin D1 & Bevacizumab D1,15 × 4 cycles (cycle = 28 days) → ddAC × 4 cycles (cycle = 14 days) & Bevacizumab D1 × 2 cycles (cycle = 14 days)
						>4 weeks postoperative: Bevacizumab D1, 15 × 8 cycles (cycle = 28 days)
NCT00528567	Recruiting	Hoffmann-La Roche; International	Phase III	Adjuvant	Operable primary invasive breast cancer	Arm A: Standard adjuvant chemotherapy (anthracycline ± taxane or taxane only) & 1 yr of Bevacizumab 5 mg/kg/week dosing equivalent
						Arm B: Standard adjuvant chemotherapy (anthracycline ± taxane or taxane only)
NCT00887575	Recruiting	Tennessee Oncology, PLLC (Nashville, TN)	Phase I/II	Neoadjuvant	T1-3, any N (excluding T1N0)	Paclitaxel D1, 8, 15 & Carboplatin D1 & Sunitinib D1-21 × 6 cycles (cycle = 28 days)
NCT01194869	Recruiting	Emory University (Atlanta, GA)	Phase II	Neoadjuvant	I-IIIA	Sorafenib 400 mg BID throughout the study: single agent for weeks 1–4, then in combination with cisplatin followed by dose dense paclitaxel
*Poly(ADP-ribose) polymerase (PARP) inhibitors*						
NCT00813956	Recruiting	Stanford Comprehensive Cancer Center (Stanford, CA)	Phase II	Neoadjuvant	I-IIIA	Gemcitabine & Carboplatin & BSI-201 q3wks
NCT01204125	Recruiting	Grupo Espanol de Estudio Tratamiento y Otras Estrategias Experimentales en Tumores Solidos (France/Spain)	Phase II	Neoadjuvant	II-IIIA	Arm A: Iniparib 5.6 mg/kg D1, 4 &Paclitaxel 80 mg/m^2^ D1 weekly × 12 weeks
						Arm B: Iniparib 11.2 mg/kg D1 &Paclitaxel 80 mg/m^2^ D1 weekly × 12 weeks
						Arm C: Paclitaxel 80 mg/^2^ D1 weekly × 12 weeks
NCT01074970	Recruiting	Indiana University Melvin and Bren Simon Cancer Center (Indianapolis, IN)	Phase II	Adjuvant	Residual disease post neoadjuvant chemotherapy (I-III)	Arm A: PF-01367338 D1-3 C1: 30mg C2-4: 24 mg & Cisplatin 75 mg/m^2^ D1 × 4 cycles (cycle = 21 days)
						Arm B: Cisplatin 75 mg/m^2^ D1 × 4 cycles (cycle = 21 days)

*EGFR inhibitors*						
NCT01097642	Recruiting	The Methodist Hospital Research Institute (Houston, TX)	Phase II	Neoadjuvant	T1N1-3M0 or T2-4 N0-3M0	Arm A: Cetuximab 400 mg/m^2^ D1 then weekly 250 mg/m^2^ & Ixabepilone 40 mg/m^2^ D1 1 × 4 cycles (cycle = 21 days)
						Arm B: Ixabepilone 40 mg/m^2^ D1 1 × 4 cycles (cycle = 21 days)
NCT00600249	Recruiting	Centre Jean Perrin (Clermont-Ferrand, France)	Phase II	Neoadjuvant	II-IIIa	Cetuximab Wk1D1 400 mg/m^2^ → 250 mg/m^2^ D1 weeks 2–18. * *& Docetaxel 100 mg/m^2^ D1 × 6 cycles (cycle = 21 days)
NCT00491816	Active/Not recruiting	University of Kansas Medical Center (Kansas City, KS)	Phase II	Neoadjuvant	II-III (T2-4, N1-2)	Erlotinib150 mg orally D3-14 with cycles 1 to 6 or 3 to 6 of neoadjuvant chemotherapy. Adjuvant chemotherapy given at the discretion of treating physician followed by 1 yr of maintenance erlotinib 150 mg daily

*Other targeted agents*						
NCT00930930	Recruiting	Vanderbilt-Ingram Cancer Center (Nashville, TN)	Phase II	Neoadjuvant	II-III	Arm A: Cisplatin & Everolimus D1 weekly × 12 weeks & Paclitaxel D1 weekly in weeks 4–12
						Arm B: Cisplatin & Placebo D1 weekly × 12 weeks & Paclitaxel D1 weekly in weeks 4–12
NCT00499603	Active/Not recruiting	M.D. Anderson Cancer Center (Houston, TX)	Phase II	Neoadjuvant	IIa-IIIc	Arm A: Drug: Paclitaxel 80 mg/m^2^ D1 weekly & RAD001 30 mg D1, 8, 15 × 12 cycles (cycle = 21 days) → 5-Fluorouracil 500 mg/m^2^ & Epirubicin100 mg/m^2^ & Cyclophosphamide 500 mg/m^2^ D1 × 4 cycles (cycle = 21 days)
						Arm B: Paclitaxel 80 mg/m^2^ D1 weekly × 12 cycles (cycle = 21 days) → 5-Fluorouracil 500 mg/m^2^ & Epirubicin100 mg/m^2^ & Cyclophosphamide 500 mg/m^2^ D1 × 4 cycles (cycle = 21 days)

*Details outlined above as per http://clinicaltrials.gov/; accessed February 28th, 2011.
